# An Automated HIV-1 Env-Pseudotyped Virus Production for Global HIV Vaccine Trials

**DOI:** 10.1371/journal.pone.0051715

**Published:** 2012-12-27

**Authors:** Anke Schultz, Stefanie Koch, Martina Fuss, Angela S. Mazzotta, Marcella Sarzotti-Kelsoe, Daniel A. Ozaki, David C. Montefiori, Hagen von Briesen, Heiko Zimmermann, Andreas Meyerhans

**Affiliations:** 1 Fraunhofer Institute for Biomedical Engineering, St. Ingbert, Germany; 2 Department of Virology, Saarland University, Homburg, Germany; 3 Department of Surgery, Duke University Medical Center, Durham, North Carolina, United States of America; 5 ICREA Infection Biology Laboratory, Department of Experimental and Health Sciences, Universitat Pompeu Fabra, Barcelona, Spain; Institut Pasteur, France

## Abstract

**Background:**

Infections with HIV still represent a major human health problem worldwide and a vaccine is the only long-term option to fight efficiently against this virus. Standardized assessments of HIV-specific immune responses in vaccine trials are essential for prioritizing vaccine candidates in preclinical and clinical stages of development. With respect to neutralizing antibodies, assays with HIV-1 Env-pseudotyped viruses are a high priority. To cover the increasing demands of HIV pseudoviruses, a complete cell culture and transfection automation system has been developed.

**Methodology/Principal Findings:**

The automation system for HIV pseudovirus production comprises a modified Tecan-based Cellerity system. It covers an area of 5×3 meters and includes a robot platform, a cell counting machine, a CO_2_ incubator for cell cultivation and a media refrigerator. The processes for cell handling, transfection and pseudovirus production have been implemented according to manual standard operating procedures and are controlled and scheduled autonomously by the system. The system is housed in a biosafety level II cabinet that guarantees protection of personnel, environment and the product. HIV pseudovirus stocks in a scale from 140 ml to 1000 ml have been produced on the automated system. Parallel manual production of HIV pseudoviruses and comparisons (bridging assays) confirmed that the automated produced pseudoviruses were of equivalent quality as those produced manually. In addition, the automated method was fully validated according to Good Clinical Laboratory Practice (GCLP) guidelines, including the validation parameters accuracy, precision, robustness and specificity.

**Conclusions:**

An automated HIV pseudovirus production system has been successfully established. It allows the high quality production of HIV pseudoviruses under GCLP conditions. In its present form, the installed module enables the production of 1000 ml of virus-containing cell culture supernatant per week. Thus, this novel automation facilitates standardized large-scale productions of HIV pseudoviruses for ongoing and upcoming HIV vaccine trials.

## Introduction

The Human Immunodeficiency Virus (HIV) continues to threaten human health. In the year 2010, around 34 million people were living with HIV as reported by the world health organization (UNAIDS World AIDS Day Report, 2011; www.unaids.org). Despite significant advances in antiviral therapy, difficulties at eradicating the latent viral reservoir, development of drug resistance as a consequence of virus hypervariability and limited access to optimal treatment due to tremendous costs are major obstacles for an effective control of the pandemic [1], [2], [3]. A preventive HIV vaccine is therefore considered essential for long-term improvements [4]. However, until today only three human clinical vaccine trials of phases IIb and III have been accomplished. The first candidate AIDSVAX consisted of recombinant HIV gp120. It was aimed to stimulate protective antibody responses, however it completely failed to prevent infections [5]. The MRKAd5 vaccine of the STEP and Phambili phase IIb trials consisted of an attenuated adenovirus 5 with inserted *gag*, *pol* and *nef* genes. Both trials were stopped in September 2007 due to enhancement of HIV infections rather than control [6]. Finally, the phase III study in Thailand (RV144) used a combination of a recombinant canarypox vector vaccine (ALVAC) and booster injections with recombinant glycoprotein 120 subunit vaccine (AIDSVAX). It induced, with 31.2% efficacy, partial protection [7]. Although this effect was only modest, it was the first demonstration of a protective impact of an HIV vaccine candidate in humans. Moreover, the low level of efficacy observed provided a way to assess whether neutralizing antibodies play an important role in vaccine-induced protection [8], [9]. In line with these findings are recent isolations of broadly neutralizing antibodies (bNAbs) PG9 and PG16 [10], [11], the very potent PGT series of bNAbs of elite neutralizers [12] and other bNAbs, as well as models on how to trigger them in humans [13], [14].

Neutralization assays are the common tool for evaluating neutralization profiles of HIV-infected or vaccinated individuals. A number of different assays with variable advantages and limitations are available (for summary see NeutNet Report [15]). One of these assays measures the antibody-mediated neutralization of HIV pseudoviruses in TZM-bl cells (also known as JC53BL-13), a genetically modified HeLa cell line that stably expresses HIV receptors and the firefly luciferase reporter gene under the control of the HIV long-terminal repeat. The TZM-bl assay allows the quantitative measurement of single-round HIV infection by HIV env-pseudotyped viruses (pseudoviruses) as Tat-dependent luciferase activity [16], [17]. In the presence of neutralizing antibodies, the infection of the indicator cells is inhibited and thus luciferase activity is reduced. By using serial dilutions, the inhibitory potential of an antibody preparation or a patient serum can be specified as ID50, the inhibitory dose to achieve 50% virus neutralization. The use of pseudovirus panels with defined HIV envelope sequences from different sources enables to analyze the breadth of the antibody response and helps to subsequently derive antibody structure/function relationships [18], [19]. Other advantages of the high throughput TZM-bl assay are the ease of handling TZM-bl cells and the defined nature of the HIV pseudoviruses. Together these aspects favor the TZM-bl assay as one of the “gold standard” neutralization tests in HIV vaccine trials [20], .

In order to cover the increasing demand of high quality reagents for ongoing and upcoming HIV vaccine trials, we established an automated system for production of HIV-1 Env-pseudotyped viruses. The automation includes stable cultivation of the cell line 293T/17 that is used for cell transfection and virus production. It offers the advantage of liter-scale, operator-independent and consistent virus production under GCLP guidelines. This automated HIV pseudovirus production and distribution to GCLP-compliant, international test laboratories [37] will help to overcome inter-laboratory differences noticed in the HIV pseudovirus stock preparations [23], [24] and thus help to improve the comparability of neutralizing antibody tests in HIV vaccine trials.

## Results

### Implementation of an automated system for cell cultivation and HIV-1 pseudovirus production

**Figure 1:**
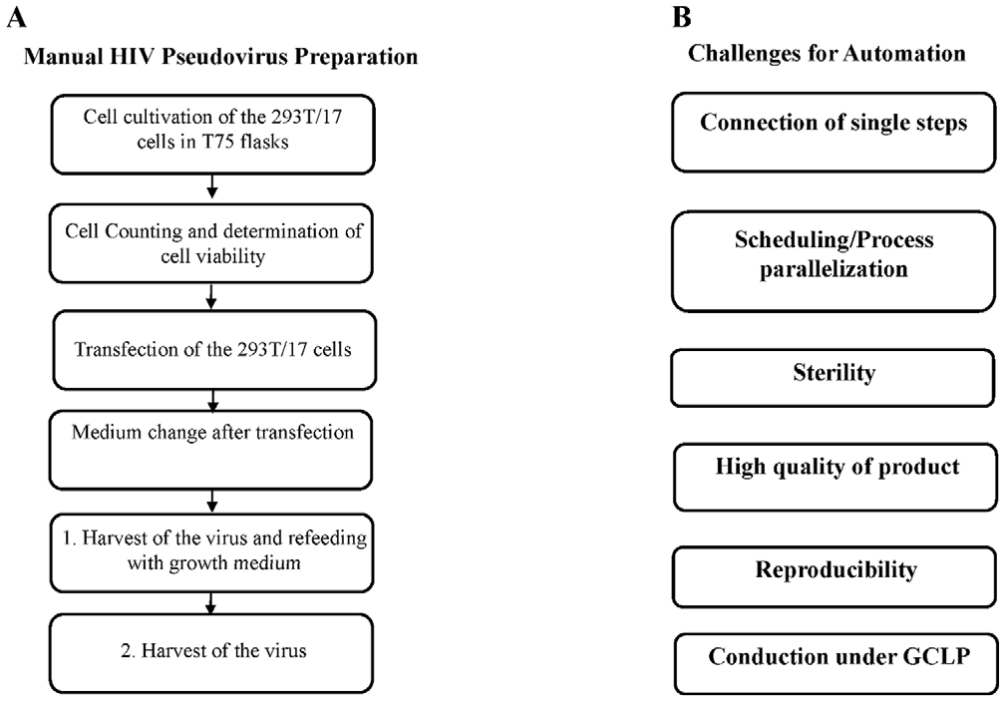
Key experimental steps in HIV pseudovirus production and challenges for automation. (A) Summary of the key experimental steps of the manual procedure for the production of HIV-1 Env-pseudotyped viruses. (B) Definition of the main challenges to transfer the manual process to the automated system.

**Figure 2:**
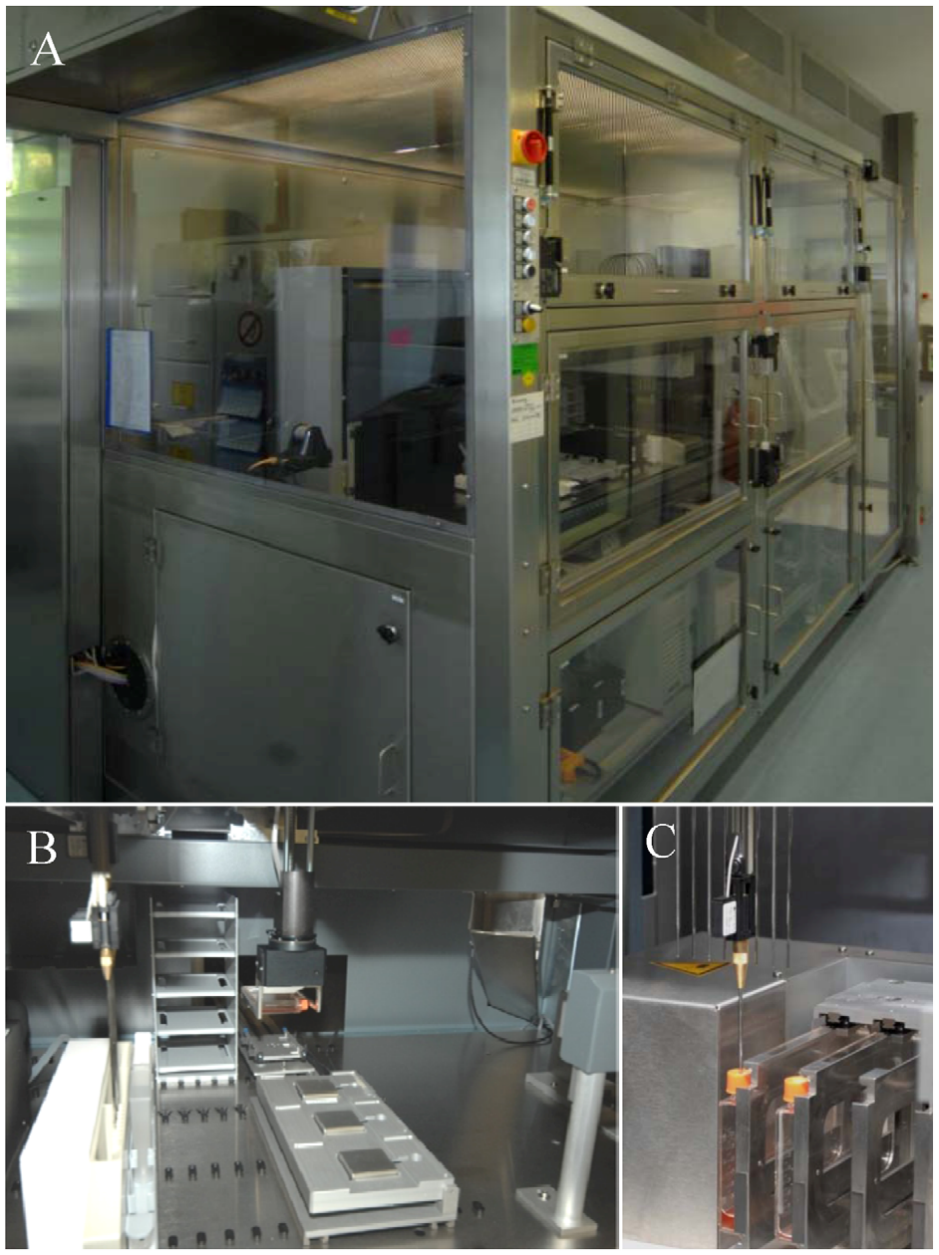
Automated system for cell culture maintenance and HIV pseudovirus production. (A) The complete system covered with a biosafety cabinet class II. (B) The robotic manipulator arm (RoMa) transports RoboFlasks from the incubator to the Flask Flipper at the worktable. (C) 2 RoboFlasks are fixed at the Flask Flipper and pierced by the needle of the Liquid Handling Arm (LiHa) for aspirating or dispensing liquids.

To cover the worldwide demand of high quality HIV-1 Env-pseudotyped viruses for antibody assessment in HIV vaccine trials, an automated production system was established. The main challenge of transferring the clearly defined steps of the manual procedure to an automated system was to combine the necessary hardware requirements with a computerized system enabling the scheduling and the connection between the single steps (Figure 1). Furthermore, sterile production conditions and a reproducible quality of the produced pseudovirus stocks had to be ensured. Finally the automated production was aimed to be conducted under GCLP-conditions due to the intended use for HIV vaccine trials. To combine these requirements a modified Tecan-based Cellerity™ system (Tecan) for cell culture maintenance and transfection has been developed (Figure 2A). The core of the system is a standard Freedom EVO 200 platform which is a modular build system for cell cultivation and handling. The robotic manipulator arm (RoMa) handles the automation friendly cell culture flasks (RoboFlasks®) within the system mediating the transport from the transfer station to the Flask Flipper and vice versa (Figure 2B). The transfer station is located on the worktable which is connected to the flask transport bridge of the CO_2_ incubator Storex (Liconic) where the RoboFlasks with 293T/17 cells are incubated at 37°C, 5% CO_2_ and 95% humidity at specified barcode-defined positions. The Flask Flipper shown in figure 2C serves as a rack for a maximum of 4 RoboFlasks in horizontal as well as in vertical position and enables the liquid handling and all pipetting actions via the liquid handling arm (LiHa) by piercing the septum of the RoboFlasks with the 8 reusable steel needles. The included shaking and knocking function of the Flask Flipper support the distribution of reagents inside the flasks and the detachment of the adherent 293T/17 cells from the flask bottom. Trypsin-EDTA and PBS, the reagents needed for the detachment of 293T/17 cells, are provided on the worktable in defined carriers and vessels. These reagents are aspirated and dispensed with built-in 5 ml syringes. The other reagents like water, disinfectant and medium are stored in a refrigerator (Revco) that is connected to the system via tubes. They are introduced into the system via the peristaltic pump or the controlled pump option (CPO). To ensure sterile conditions, the worktable is housed in a biosafety class II cabinet (Laminar Flow Hood Type WK13.33, BDK) that protects personnel, product and environment. In addition, the tubing system is cleaned after each cell maintenance task and each production run with 0.4% Peraclean (Degussa) within the program Clean System (2 min with 0.4% Peraclean and 10 min with sterile water). In manual experiments, imitating the disinfection process running on the automated system, we confirmed the disinfection efficiency with respect to 293T/17 cells and inactivation of HIV-1 pseudoviruses (data not shown).

The linkage between the hardware components and the automatically performed actions is controlled by two software programs. The Freedom Evoware Plus Software controls the RoMa, the LiHa, the Flask Flipper and the transfer station. This software is also used to define and program all single steps of the individual processes. The CellGEM (Cell Growth, Expansion, Maintenance) software is of overriding importance and is responsible for scheduling the time for splitting cells, harvesting and management of cell culture parameters, disposables and reagent availability. Cell culture processes (maintenance request) or pseudovirus production (production request) were established using this software.

**Figure 3:**
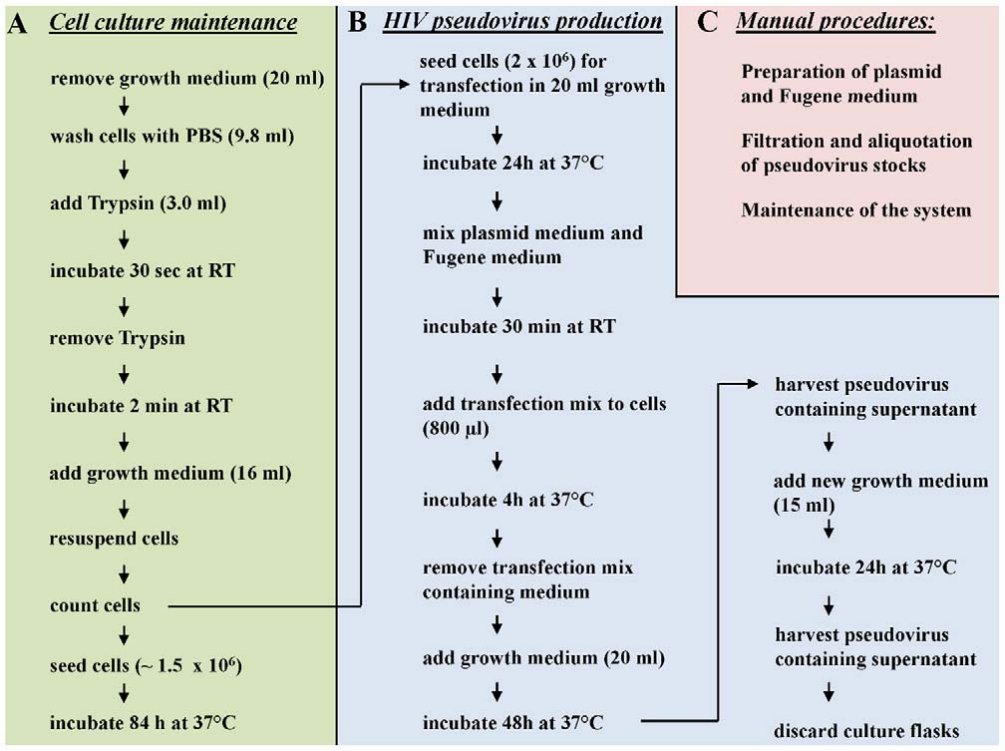
Detailed workflow of the automated procedure. (A) Cultivation of the 293T/17 cells and (B) the HIV-1 pseudovirus preparation with the adapted volumes of growth medium, PBS, Trypsin-EDTA, the incubation times and the cell numbers. (C) The performed manual steps.

A major issue in the overall automation procedure was the exact enumeration of viable cells. This was solved by incorporating a Cedex Cell Counter (Roche, Innovatis), a validated automated cell counting system [25], under the control of the Freedom Evoware software. The Cedex Cell Counter functions with the principle of Trypan blue exclusion staining and records microscopic pictures that are analyzed by an inherent imaging software. The calculation of the number of available cells and the doubling time based on the results measured with the Cedex Cell Counter are necessary parameters for the automated cell culture. With this information the CellGEM software defines how many cells need to be seeded to reach the expected cell number per RoboFlask (between 6×10^6^ to 30×10^6^ cells) at the next split date. In general, the seeded cell numbers range from 1.5×10^6^ to 3×10^6^. The exact amounts of reagents, time points and cell numbers are the results of manual experiments during the optimization phase of the automation. Suitability of the selected parameters for HIV-1 Env-pseudotyped virus stock production was verified in subsequent neutralization assays with defined test reagents (Table S1). The workflow for cell cultivation and HIV pseudovirus production is summarized in figure 3.

### Stable cultivation of the 293T/17 cells on the automated system

**Figure 4:**
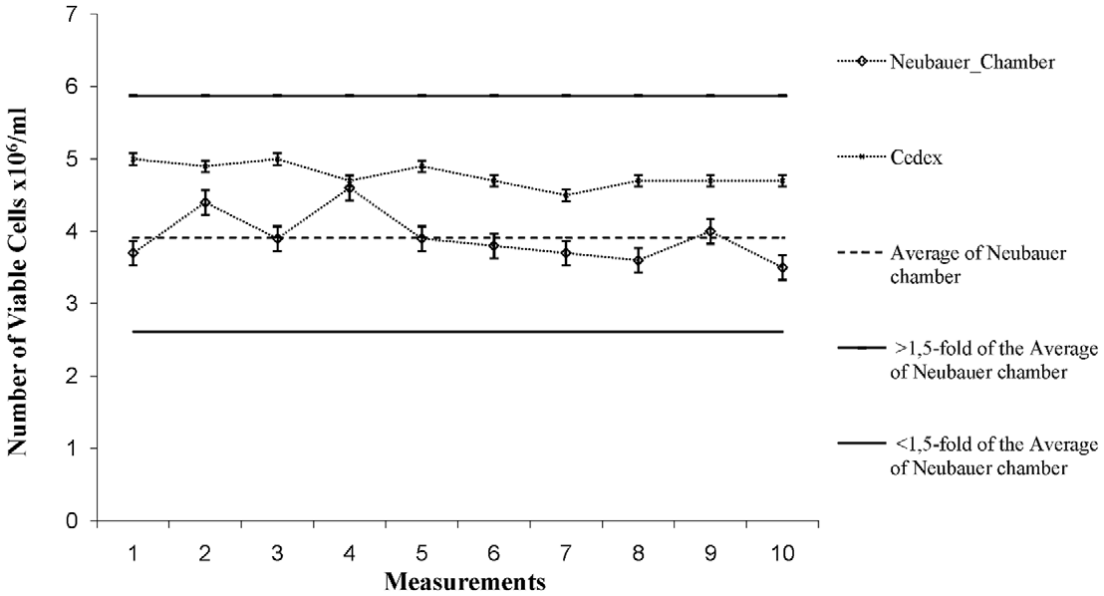
Accuracy of the measurements of cell density. Illustrated are 10 measurements of a suspension of 293T/17 cells conducted by the Cedex Cell Counter and the Neubauer hemacytometer chamber. The values of Cedex counting are within 1.5-fold of the mean value of the measurements with the Neubauer hemacytometer chamber.

The consistent quality of the cell culture within the automated system is an important criterion for the reliable supply of 293T/17 cells for HIV-1 pseudovirus production. Therefore the accurate determination of cell numbers with the Cedex Cell Counter is necessary and has been verified with ten parallel measurements of a suspension of 293T/17 cells by the Cedex Cell Counter and the Neubauer hemacytometer chamber. The results demonstrated that the cell concentrations measured by the Cedex Cell Counter never deviated more than 1.5-fold from the mean value determined with the Neubauer hemacytometer chamber thus meeting pre-defined acceptance criteria (Figure 4). Moreover the accuracy of the Cedex Cell counter was also tested with control beads (Roche) of certified particle concentrations (5×10^5^/ml, 1×10^6^/ml, and 5×10^6^/ml) in triplicate measurements for each density. The results were within the allowed 7.5% deviation from baseline as defined by the manufacturer. The baseline was defined by 10 measurements of control beads (data not shown).

**Figure 5:**
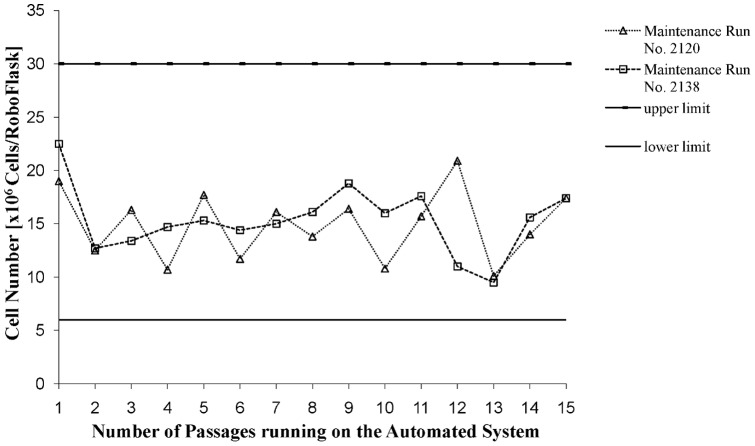
Stable cell number of 293T/17 cells cultivated with the automated system. Shown are 2 different maintenance runs (No. 2120, No. 2138) cultivated for 15 passages with the automated system. The cell numbers range for each splitting task between the ranges of 6×10^6^ and 30×10^6^ cells per harvested RoboFlask.

Before the 239T/17 cells could be used for pseudovirus preparation, stable cell growth, cell maintenance and cell viability had to be demonstrated on the automated system. For this, two independent maintenance runs were tracked over 15 passages. The number of viable cells per RoboFlask varied in request number 2120 from 10.1 to 20.9×10^6^ and in request number 2138 from 9.5 to 22.5×10^6^. This was well within the pre-defined limits of 6 to 30×10^6^ cells per RoboFlask (Figure 5). To test the cell viability under the defined culture conditions, four independent cell culture maintenance tasks were run. The overall viability of the 293T/17 cells ranged between 91.1% and 99.1% (Table 1) thus demonstrating robust cell cultivation with the process parameters used.

**Table 1:** Viability of 293T/17 cells maintained over 6 passages on the automated system.

Maintenance Run No.	Passage Number	Cell viability in (%)
2120	18–24	91.9–96.6
2121	20–26	91.8–96.4
2127	34–40	93.6–97.5
2135	39–45	94.3–97.8

### Accuracy and Reproducibility of the automated production of HIV-1 Env-pseudotyped viruses

To prepare HIV-1 pseudoviruses on the automated system, the following specific scripts with defined parameters were implemented: (I) Delivery Harvesting Automation (harvest of the 293T/17 cells and the collection in a vessel), (II) Delivery Transfer (seeding of 2×10^6^ 293T/17 cells per RoboFlask), (III) Post Delivery Transfer (transfection) and (IV) Medex2 for delivery (harvest of the virus containing supernatant). In 5 different small-scale production tasks (140 ml each) the settings were established on the automated system. The quality of the automated production of small-scale pseudovirus stocks consisting of 6535.3 (tier 1B), QH0692.42 (tier 2) and PVO.4 (tier 3) all classified as HIV-1 Clade B viruses [26] and SF162.LS and MN.3, two Clade B tier 1A viruses [27], [28] were evaluated in parallel neutralizing antibody assays (bridging tests) with manually prepared reference stocks using defined test reagents (sCD4, IgG1b12, 2F5, 4E10 and TriMab). All neutralization titers were within 1/3-fold to 3-fold of their mean value (Table S2). Therefore, the acceptance criteria, defining that 80% of the test reagents must be within 3-fold of the mean value were satisfied. Subsequently, the accuracy of a large-scale production with an increased volume of up to 1000 ml per virus stock was tested. For this, three Clade B tier 2 viruses (REJO4541.67, QH0692.42, WITO4160.33), one Clade B tier 1A virus (SF162.LS) and one Clade C tier 1B virus (ZM197M.PB7) [21], [26], [27], [28] were prepared with the automated system. The bridging tests revealed concordant neutralization titers within the acceptance criteria of 1/3-fold to 3-fold of their mean value (Table 2). This demonstrated large-scale pseudovirus production of high quality.

**Table 2:** Quality control of the precision of large-scale HIV-1 pseudovirus stocks: Comparison of automated and manual produced viruses by neutralization titers with test reagents.

	ID50 values (µg/ml) of virus stocks determined with HIV-neutralizing test reagents				
Pseudovirus	sCD4	IgG1b12	2F5	4E10	TriMab
HIV-REJO4541.67 automate	1.79	2.84	0.98	2.70	1.37
HIV-REJO4541.67 manual	1.89	2.71	1.25	2.70	1.54
HIV-QH0692.42 automate	3.94	1.13	6.44	19.33	1.33
HIV-QH0692.42 manual	3.10	0.96	2.49	7.14	0.89
HIV-ZM197M.PB7 automate	14.23	>25	44.28^a^	1.13	31.46^a^
HIV-ZM197M.PB7 manual	11.56	24.41	29.94^a^	0.98	27.07^a^
HIV-WITO4160.33 automate	9.36	8.76	2.13	2.82	1.95
HIV-WITO4160.33 manual	9.00	7.20	3.45	4.63	3.29
HIV-SF162.LS automate	0.14	0.04	0.14	8.95	2.83
HIV-SF162.LS manual	0.13	0.03	0.10	5.52	1.42

a initial concentration 50 µg/µl.

In order to determine the reproducibility of the automated pseudovirus production, 5 virus stocks were prepared consecutively on the automated system using the same plasmid stocks for both backbone plasmid (pSG3Δenv) and env plasmid (SF162.LS). These preparations were compared to the preparation of a single virus stock produced with the manual procedure using the same backbone and env plasmids used in the automated production. Pseudovirus SF162.LS was chosen because it is used in neutralization experiments for HIV vaccine development due to its high neutralization sensitivity [27], [29]. Bridging the automated and manual prepared viruses against a historical SF162.LS reference preparation demonstrated that the neutralization titers of all the different SF162.LS virus stocks were within the 1/3-fold to 3-fold ID_50_ range (Table 3). Identical results were obtained when using plasma samples from HIV-1 Clade B-infected individuals as bridging test reagents (data not shown).

**Table 3:** Quality control of the reproducibility: Comparison of the neutralization titers with test reagents of automated and manual produced HIV-1 pseudoviruses.

	ID50 values (µg/ml) of virus stocks determined with HIV-neutralizing test reagents				
Pseudovirus	sCD4	IgG1b12	2F5	4E10	TriMab
HIV-SF162.LS reference stock 1. harvest	0.14	0.05	3.46	5.58	0.09
HIV-SF162.LS manual 1. harvest	0.19	0.08	5.31	12.80	0.17
HIV-SF162.LS manual 2. harvest	0.16	0.05	3.06	6.83	0.09
HIV-SF162.LS automate 1. harvest (batch 1)	0.18	0.05	2.85	8.06	0.11
HIV-SF162.LS automate 2. harvest (batch 1)	0.17	0.04	2.68	5.23	0.11
HIV-SF162.LS automate 1. harvest (batch 2)	0.18	0.05	4.52	8.50	0.13
HIV-SF162.LS automate 2. harvest (batch 2)	0.16	0.05	2.64	5.70	0.09
HIV-SF162.LS automate 1. harvest (batch 3)	0.17	0.05	3.95	7.85	0.15
HIV-SF162.LS automate 2. harvest (batch 3)	0.14	0.04	2.57	5.48	0.11
HIV-SF162.LS automate 1. harvest (batch 4)	0.16	0.06	4.08	10.96	0.18
HIV-SF162.LS automate 2. harvest (batch 4)	0.19	0.05	3.73	6.08	0.11
HIV-SF162.LS automate 1. harvest (batch 5)	0.20	0.06	4.60	10.79	0.13
HIV-SF162.LS automate 2. harvest (batch 5)	0.17	0.05	3.21	8.99	0.12
Mean of the automated productions	0.17	0.05	3.48	7.76	0,12

### GCLP-compliant automated HIV-1 pseudovirus production

The validation of the automated production procedure together with the associated components included the parameters accuracy, specificity, robustness and precision. Because accuracy and precision of the automated cell cultivation are linked to the reliable determination of viable cell numbers and consequently the reproducible production of HIV-1 pseudoviruses, the accuracy and precision of the pipetting volumes were verified by gravimetrical measurements using a SAG 285/01 balance from Mettler Toledo. Results of the 10 measurements of selected volumes (100 µl, 800 µl, 2500 µl and 4500 µl) met the pre-defined limits of accuracy (deviation to the target volume divided by the target volume) ≤7.0% and coefficient of variation (CV) ≤5% (data not shown). The robust and sterile maintenance conditions for cell cultivation was evaluated with 293T/17 cells maintained on the system in antibiotic-free sterile medium for 6 passages (3 weeks). Bacteria and fungi tested negative in a bioburden assay using Tryptic Soy Agar plates according to the manufacturer (heipha Dr. Müller GmbH, Eppelheim, Germany). Likewise, tests for Mycoplasma contamination by a third party testing facility (Labor Dr. Thiele, Institut für Immunologie und Genetik, Kaiserslautern, Germany) were negative, demonstrating the quality of the culture environment (data not shown). Furthermore, the system sterility was evaluated with sterile medium without antibiotics aspirated from the worktable by each of the eight channels and dispensed into 8 RoboFlasks. After 7 days at 37°C/5% CO_2_ and subsequent examination for microbial growth and cell growth by microscopic analysis, no contamination inside the 8 RoboFlasks was detected. Thus the sterilization procedure of the tubing and needles with 0.4% Peraclean solution within the Clean System program at the end of each process was successfully validated.

The created standard operating procedures (SOPs) directing all the operations of the automated system including cell cultivation and pseudovirus production, as well as the associated components, equipment and reagent preparations in a GCLP-compliant manner were formally audited by the Central Quality Assurance Unit (CQAU) of CAVD/CA-VIMC and externally audited by PPD Laboratory Services and Sailstad & Associates. With this, the established automated HIV-1 pseudovirus preparation system is ready to produce high quality reagents for worldwide vaccine trials.

## Discussion

Here we present the results of the transfer of a manual production procedure for HIV-1 pseudoviruses to an automated system in a GCLP-compliant manner. This automated system consists of a modified Tecan-based Cellerity system and performs the complete production process with several dependent steps under computer control. It allows the accurate production of liter-scale pseudovirus stocks with concordant quality. In addition, the reproducibility and robustness of the production procedure was demonstrated by the generation of 5 batches of the Clade B pseudovirus HIV-SF162.LS using the same plasmid stocks. Besides the independency from external influences, the advantage of the automated system is the day and night operation that allows the stable and scalable supply of the producer cell line 293T/17. Other beneficial outcomes of this automated method are the robust and sterile production conditions with minimal manual interactions such as the provision of the transfection mix and the weekly maintenance. The automatic monitoring of the complete production process via the generated maintenance reports facilitates the traceability of all individual steps. In addition, an email notification system has been established to inform the personnel about state change, depletion of resources and potential errors so that necessary steps can be initiated immediately. Furthermore, the temperatures of the refrigerator, worktables and reagents troughs are tracked automatically in order to guarantee the stability of the production environment. All electronic information is saved on a server for long-term storage and retrieval if need occurs. Given this functional prototype automation, procedure modifications to produce other reagents including replication competent viruses like those with incorporated marker genes [30] are now easily possible.

Automated cell cultivation plays an increasing role in numerous fields of science providing the advantage of removing process variability due to operator dependency and reducing occupation of personnel with repetitive routine tasks [31]. In particular the unattended operation over night and weekend allows the delivery of a certain cell type in flexible formats (microtiter plates, flasks etc.). Various cell types including primary cells, adherent and non-adherent mammalian cell lines can be maintained automatically and expanded for assays where large quantities of cells are needed [32], [33]. Therefore, the existing applications extend from the automated maintenance of cells in drug discovery programs to the high-throughput supply of manufacturing cell lines for monoclonal antibody production [34]. Besides this, great efforts are made to promote the automation of the differentiation and cultivation of embryonic stem cells (ESC) with the aim to reduce their labor-intensive maintenance as well as their large-scale production [35], [36], [37]. Finally, the recently described automated transfection of mammalian cells for protein expression between 10 and 200 kDa [38] emphasizes the importance of automated platforms in the standardized production process of biological reagents. However, one should bear in mind that such platforms have to be validated under some quality regulations when products were to be used as therapeutics or in vaccine trials.

For the automation described here, GCLP standards [39], [40] were selected for validation because they are the interface between the requirements of the Good Laboratory Practice (GLP), limited to non-clinical studies and the Good Clinical Practice (GCP), which focus much less on basic, non-diagnostic laboratory results. Furthermore, the GCLP-compliant methods ensure that the results are reliable, reproducible, auditable and comparable between preclinical studies, clinical trials and manufacturing [23], [41]. Therefore, in the optimization period the acceptance ranges as well as assay conditions that may affect the method parameters were determined. Based on these results, a validation plan was developed in which the specific criteria for the validation parameters accuracy, precision, specificity and robustness were defined, acceptance criteria were pre-established, based on ICH Q2A and Q2(R1) guidelines (http://www.ich.org) and the plan was officially authorized by the quality assurance unit. The experiments described in this article are the key findings of the validation protocol and provide the documented evidence that the automated system operates accurately and consistently. The automated method as well as the related documents and SOPs were constantly audited by the Central Quality Assurance Unit and its independent consultant auditors, which in the end resulted in the full validation of the automated process.

In conclusion, we have assembled a complete, GCLP-validated, automated unit for reliable cell cultivation, transfection and HIV pseudovirus production of high quality. Its large production scale combined with its high reproducibility will facilitate reagent production for ongoing and upcoming HIV vaccine trials. As the overall set-up of this prototype system is very flexible, other applications involving cell cultivation, transfection and harvesting of supernatants may easily be implemented. Thus this system holds great promise for future production of biological reagents of diverse applications.

## Materials and Methods

### Cells

The TZM-bl cell line was obtained through the NIH AIDS Research and Reference Reagent Program (ARRRP, catalog no. 8129), as contributed by J. Kappes and X. Wu [42]. It is derived from a genetically engineered HeLa cell clone that expresses CD4, CXCR4, and CCR5 and contains Tat-responsive reporter genes for firefly luciferase (Luc) and *Escherichia coli* ß-galactosidase under regulatory control of an HIV-1 long terminal repeat [16], [17]. 293T/17 cells were obtained from LGC Promochem (ATCC-CRL-11268). Both cell lines were maintained in growth medium composed of Gibco's Dulbecco's modified Eagle's medium (Life Technologies, Paisley, UK) containing 10% heat-inactivated fetal bovine serum (FBS), 25 mM HEPES and 50 µg/ml gentamicin in vented T-75 culture flasks (Greiner bio-one) or in RoboFlasks® (Corning) for the automated cell cultivation. Cultures were incubated at 37°C in a humidified 5% CO_2_–95% air environment. Cell monolayers were split by treatment with 1x Trypsin-EDTA (1∶250, PAA).

### HIV-1 pseudovirus production and titration

The manual HIV pseudovirus production procedure in T-75 culture flask was described previously [43]. For the automated large-scale production of HIV pseudoviruses (30 RoboFlasks) 2×10^6^ cells per flask were seeded in 20 ml growth medium. After 24 h of incubation the cells were transfected with 4 µg of a HIV-1 *env* expression plasmid and 8 µg of an *env*-deficient HIV-1 backbone vector (pSG3ΔEnv), using Fugene®6 transfection reagent (Roche). The transfection complexes were incubated for 30 min at room temperature before distribution to 16 RoboFlasks (800 µl each) within four serial “Transfection_4” processes. For transfection of the remaining 14 RoboFlasks the described procedure is repeated. After 4 h of incubation at 37°C, 5% CO_2_–95% humidity atmosphere, the transfection medium is removed from the flasks and new medium (20 ml each) is added. After incubation at 37°C, 5% CO_2_ and 95% humidity the pseudovirus containing culture supernatants were harvested 48 h (20 ml per RoboFlask) and 72 h (15 ml per RoboFlask) after transfection, filtered (0.45 µm) and stored at −80°C in 1 ml aliquots.

The 50% tissue culture infectious dose (TCID) of a single thawed aliquot of each batch of pseudovirus was determined in TZM-bl cells as described elsewhere [43]. For TCID measurements, serial 5-fold dilutions of pseudovirus were made in quadruplicate in 96-well culture plates in a total volume of 100 µl of growth medium for a total of 11 dilution steps. Freshly trypsinized cells (1×10^4^ cells in 100 µl of growth medium containing 10 µg/ml DEAE-Dextran) were added to each well, and the plates were incubated at 37°C in a humidified 5% CO_2_–95% air environment. After 48 h incubation, 100 µl of culture medium was removed from each well and 100 µl of britelite™ plus reagent (Perkin Elmer) was added to the cells. After 2 min incubation at room temperature, 150 µl of cell lysate was transferred to 96-well black solid plates (Corning®-Costar®) for measurements of luminescence using a Infinite® F200 luminometer (Tecan). Wells with virus containing supernatant that is not toxic to the cells based on light microscopy inspection and that produces relative luminescence units (RLU) >10 times background were scored as positive. The TCID was calculated as described [44].

### Neutralizing Ab assay

Neutralizing antibodies were measured as reductions in Luciferase reporter gene expression after a single round of virus infection of TZM-bl cells as described previously [19], [24]. Briefly, 3-fold serial dilutions of test reagent and neutralizing antibodies (sCD4; Progenics and IgG1b12, 2F5, 4E10, TriMab; Polymun, initial concentration of 25 µg/µl) were performed in duplicate (96-well flat bottom plate) in DMEM growth medium (100 µl/well). An amount of virus to achieve a TCID of 150 000 RLU equivalents was added to each well in a volume of 50 µl. The plates were then incubated for 45–90 min at 37°C in a total volume of 150 µl growth medium. Freshly trypsinized cells (1×10^4^ cells in 100 µl of growth medium containing 10 µg/ml DEAE-Dextran) were added to each well. After 48 h incubation, 150 µl of culture medium was removed from each well and 100 µl of britelite™ plus reagent was added to the cells. After 2 min incubation at room temperature to allow cell lysis, 150 µl of the cell lysate was transferred to black, solid 96-well plates for measurements of luminescence using a Infinite® F200 luminometer. Neutralization titers (the 50% inhibitory dose (ID50)) were defined as the sample concentration (sCD4 and MAbs) that caused a 50% reduction in RLU compared to virus control wells after subtraction of background control RLU. As described previously [43], the assays were evaluated as passed when following parameters were met: the mean RLU of the virus control was ≥10x higher than the background of the cell control; the standard deviation of RLU in the virus control well was ≤30%; the standard deviation for duplicate wells was ≤30%; the neutralization curves were smooth and linear around the 50% neutralization cut-off; the value of the positive control agreed with the previous values for that particular control-virus combination. All data were analyzed with 5-parameter curve fitting using neutralizing antibody analysis software provided by the CAVD Vaccine Immunology Statistical Center.

### Validation design

Validation parameters evaluated in this study included accuracy, specificity, robustness and precision. The accuracy of the cell density was assayed using standard reference beads (Roche) with a defined concentration (10.06×10^5^/ml) and the suspension of the 293T/17 cells seeded with the density of 2×10^6^ in each of three T-75 cell culture flasks for counting after 24 h, 48 h, 72 h and 96 h. The reference beads and the cells of each flask were counted in parallel via the Neubauer hemacytometer chamber and the Cedex Cell Counter. On the basis of these results (data not shown), acceptance criteria were defined: the values measured by the Cedex Cell Counter must be within 1.5-fold compared to the Neubauer chamber.

The limit to test the accuracy and precision of the automatically produced HIV-1 Env-pseudotyped viruses was set by preparing five different virus stocks (QH0692.42, 6535.3, PVO.4, SF162.LS and MN.3) in small-scale (140 ml of each) and evaluating them with the bridging test in the form of two neutralization assays performed in parallel. The test was considered positive if the neutralization titers for at least 80% of the assayed reagents (sCD4, IgG1b12, 2F5, 4E10 and TriMab) agree within 3-fold between the data of the automatically produced pseudovirus and the manual prepared reference stock, corresponding the outcomes of the bridging tests of the small scale automated prepared pseudoviruses (Table S2). Subsequent to these results the pass criterion was defined: the neutralization titers of four of the five test reagents must be within 3-fold.

Robustness and specificity of the automated cell cultivation of the 293T/17 cells was implemented with respect to the number of harvested cells after 84 h of incubation on the automate and the viability. On the basis of the result of the maintenance run transferring the 293T/17 cells within the system over 13 passages, the number of harvested 293T/17 cells per RoboFlasks must range between 6×10^6^ and 30×10^6^ to establish a stable cell line (data not shown).

## Supporting Information

Table S1 **Comparable neutralization titers with test reagents of HIV-1 pseudoviruses produced in RoboFlasks and T-75 flasks grown viruses.**
(DOCX)

Table S2 **Comparison of the neutralization titers with test reagents of automated and manual produced HIV-1 pseudoviruses to determine the limits of accuracy and precision.**
(DOCX)
