# Supplementary material for: An Automated HIV-1 Env-Pseudotyped Virus Production for Global HIV Vaccine Trials
Source: PLoS One. 2012 Dec 27;7(12):e51715. doi: 10.1371/journal.pone.0051715 (PMC3531445; doi:10.1371/journal.pone.0051715)
Supplement: Table S1 — Comparable neutralization titers with test reagents of HIV-1 pseudoviruses produced in RoboFlasks and T-75 flasks grown viruses. (DOCX) [file pone.0051715.s001.docx]

|  | **ID50 values (µg/ml) of virus stocks determined with HIV-neutralizing test reagents** | | | | |
| --- | --- | --- | --- | --- | --- |
| **Pseudovirus** | **sCD4** | **IgG1b12** | **2F5** | **4E10** | **TriMab** |
| HIV-QH0692.42 (T75-flask) | 4.4 | 3.0 | 7.3 | 17.3 | 3.2 |
| HIV-QH0692.42 (RoboFlask)^a^ | 5.6 | 4.3 | 8.1 | >25 | 4.0 |
| HIV-PVO.4 (T75-flask) | 15.0 | >25 | >25 | 42.6 | 8.1 |
| HIV-PVO.4 (RoboFlask)^a^ | 15.1 | >25 | >25 | 40.6 | 4.3 |
| ^a^ The viruses produced in RoboFlasks are generated according to the conditions defined for the automated system. | | | | | |
