# Supplementary material for: An Automated HIV-1 Env-Pseudotyped Virus Production for Global HIV Vaccine Trials
Source: PLoS One. 2012 Dec 27;7(12):e51715. doi: 10.1371/journal.pone.0051715 (PMC3531445; doi:10.1371/journal.pone.0051715)
Supplement: Table S2 — Comparison of the neutralization titers with test reagents of automated and manual produced HIV-1 pseudoviruses to determine the limits of accuracy and precision. (DOCX) [file pone.0051715.s002.docx]

|  |  |  | |  | |  |  | |
| --- | --- | --- | --- | --- | --- | --- | --- | --- |
|  | **ID50 values (µg/ml) of virus stocks determined with HIV-neutralizing test reagents** | | | | | | | |
| **Pseudovirus** | **sCD4** | | **IgG1b12** | | **2F5** | | **4E10** | **TriMab** |
| **HIV-QH0692.42 automate** | 2.41 | | 0.85 | | 3.24 | | 9.10 | 2.50 |
| **HIV-QH0692.42 manual** | 1.96 | | 0.76 | | 2.54 | | 6.91 | 2.15 |
| **HIV-6535.3 automate** | 3.18 | | 15.04 | | 7.84 | | 4.83 | 6.00 |
| **HIV-6535.3 manual** | 2.14 | | 8.17 | | 9.60 | | 3.79 | 4.63 |
| **HIV-PVO.4 automate** | 16.15^a^ | | >50^a^ | | >50^a^ | | 30.97^a^ | 5.83^a^ |
| **HIV-PVO.4 manual** | 14.40^a^ | | >50^a^ | | >50^a^ | | 23.47^a^ | 4.67^a^ |
| **HIV-SF162.LS automate** | 0.18 | | 0.06 | | 2.67 | | 7.00 | 0.46 |
| **HIV-SF162.LS manual** | 0.15 | | 0.05 | | 1.73 | | 4.70 | 0.39 |
| **HIV-MN.3 automate** | <0.01 | | <0.01 | | 0.04 | | 0.14 | 0.01 |
| **HIV-MN.3 manual** | <0.01 | | <0.01 | | 0.04 | | 0.18 | 0.03 |
| ^a^ initial concentration 50 µg/µl |  | |  | |  | |  |  |
